# BK Polyomavirus—Biology, Genomic Variation and Diagnosis

**DOI:** 10.3390/v13081502

**Published:** 2021-07-30

**Authors:** Jacek Furmaga, Marek Kowalczyk, Tomasz Zapolski, Olga Furmaga, Leszek Krakowski, Grzegorz Rudzki, Andrzej Jaroszyński, Andrzej Jakubczak

**Affiliations:** 1 Department of General and Transplant Surgery and Nutritional Treatment, Medical University of Lublin, 20-954 Lublin, Poland; jacekf61@o2.pl; 2 Institute of Quality Assessment and Processing of Animal Products, University of Life Sciences in Lublin, 20-950 Lublin, Poland; 3 Department of Cardiology, Medical University of Lublin, 20-954 Lublin, Poland; zapolia@wp.pl; 4 Department of Radiology, 424 General Military Hospital, 56403 Thessaloniki, Greece; ola.furmaga@gmail.com; 5 Department and Clinic of Animal Reproduction, Faculty of Veterinary Medicine, University of Life Sciences, Gleboka 30, 20-612 Lublin, Poland; leszek.krakowski@up.lublin.pl; 6 Department of Endocrinology, Diabetology and Metabolic Diseases, Medical University of Lublin, 20-954 Lublin, Poland; grzegrudzki@gmail.com; 7 Department of Nephrology, Jan Kochanowski University in Kielce, 25-232 Kielce, Poland; jaroszyskiaj@interia.pl; 8 Institute of Biological Basis of Animal Production, Faculty of Animal Sciences and Bioeconomy, University of Life Sciences in Lublin, 20-950 Lublin, Poland

**Keywords:** BK polyomavirus, BK polyomavirus subtypes, molecular polymorphism, molecular diagnostics

## Abstract

The BK polyomavirus (BKPyV), a representative of the family Polyomaviridae, is widespread in the human population. While the virus does not cause significant clinical symptoms in immunocompetent individuals, it is activated in cases of immune deficiency, both pharmacological and pathological. Infection with the BKPyV is of particular importance in recipients of kidney transplants or HSC transplantation, in which it can lead to the loss of the transplanted kidney or to haemorrhagic cystitis, respectively. Four main genotypes of the virus are distinguished on the basis of molecular differentiation. The most common genotype worldwide is genotype I, with a frequency of about 80%, followed by genotype IV (about 15%), while genotypes II and III are isolated only sporadically. The distribution of the molecular variants of the virus is associated with the region of origin. BKPyV subtype Ia is most common in Africa, Ib-1 in Southeast Asia, and Ib-2 in Europe, while Ic is the most common variant in Northeast Asia. The development of molecular methods has enabled significant improvement not only in BKPyV diagnostics, but in monitoring the effectiveness of treatment as well. Amplification of viral DNA from urine by PCR (Polymerase Chain Reaction) and qPCR Quantitative Polymerase Chain Reaction) is a non-invasive method that can be used to confirm the presence of the genetic material of the virus and to determine the viral load. Sequencing techniques together with bioinformatics tools and databases can be used to determine variants of the virus, analyse their circulation in populations, identify relationships between them, and investigate the directions of evolution of the virus.

## 1. Introduction

The BK Polyomavirus (BKPyV) virus belongs to the genus Betapolyomavirus of the family Polyomaviridae [1]. Polyomaviruses are the aetiological agent of numerous diseases in people with immune deficiency caused by pathological states or immunosuppressant drugs, leading to replication of the pathogen and the development of disease. In the case of immunocompetent individuals, BKPyV infection becomes persistent, and disease symptoms are usually absent or mild.

Viruses of the family Polyomaviridae infect various species of mammals (primates and rodents) and birds [2]. The first polyomavirus, murine polyomavirus (MPyV), was isolated in 1953 by Ludwig Gross. MPyV was observed to cause adenocarcinomas of the parotid gland of newborn mice [3]. It was because of this ability to induce tumour development that the pathogen was given the name ‘polyomavirus’, from the Greek ‘poly’ (‘many’) and ‘oma’ (‘tumour’) [4]. Research confirmed that the virus was widespread in rodents, but symptoms developed only in young or immunocompromised individuals [5].

Two more representatives of the polyomaviruses were detected in the 1970s—the BK polyomavirus (BKPyV) and the JC polyomavirus (JCPyV), whose names were based on the initials of the patients they were isolated from [6,7]. BKPyV was detected in the urine of a patient who had received a kidney transplant, with failure of the transplanted kidney caused by ureteral stricture. The other virus, later named JC virus, was isolated post mortem from the brain tissue of a patient with progressive multifocal leukoencephalopathy [8,9]. There have now been 15 polyomaviruses identified in humans, most of them in the last ten years [10]. In 2007 two new polyomaviruses were identified, KI (KIPyV) and WU (WUPyV), named after the universities where they were discovered (Karolinska Institutet and Washington University). KI was detected in nasopharyngeal aspirates [11], and the presence of WU was confirmed in bronchoalveolar lavage [12]. A year later, Merkel Cell Polyomavirus (MCPyV), believed to be associated with carcinogenesis, was detected in the tissues of patients with skin tumours [13]. Other polyomaviruses detected in the skin are TSV–Trichodysplasia Spinulosa-associated polyomavirus [14], HPyV6, HPyV7 [15], and Lyon IARC PyV (LIPyV) [16]. Other viruses described in the last decade are HPyV9 [17], HPyV10 [18], QPyV [19], STLPyV [20], HPyV12 [21] and NJPyV [22]. The universal occurrence of polyomaviruses in the population was confirmed in a study of more than 1000 Dutch blood donors; antibodies against at least four different polyomaviruses were detected in all of them, and the frequency of antibodies against most polyomaviruses ranged from 60–100% [23]. Some of the detected polymoaviruses (e.g., LIPyV) are not likely to be human viruses but may be transient infections, environmental exposures or viral contaminants [16,19,23].

For more than 30 years, the JC and BK polyomaviruses were the only well-known human polyomaviruses [24]. The dynamic increase in the detection of new polyomaviruses is due to the development of numerous modern molecular methods and next generation sequencing (NGS) [3,25,26].

Interest in the BK and JC polyomaviruses is due in part to their high prevalence. Anti-BKPyV antibodies have been detected in over 80% of individuals in analysed populations, and in the case of the JC polyomavirus, antibodies indicating contact with the pathogen have been shown in about 39% of those tested [27,28,29,30]. The genetic material of the virus is detected much less often. In a study using urine samples from 164 healthy individuals, the presence of BKPyV genetic material was confirmed in nearly 13% [31]. According to the literature, in physiological conditions the virus is detected especially, in the urine of pregnant women and in elderly patients with diabetes [26].

The presence of BKPyV in organ transplant recipients most often induces urinary tract infections. In the case of kidney transplants, the disease occurs in the transplanted kidney, while in the case of transplants of other organs, such as a heart, liver or lung, BKPyV infection is observed in the recipient’s native kidneys, and occasionally in other organs [8].

BKPyV nephropathy (BKPyVAN) is the most serious complication caused by the presence of BKPyV; it can lead to loss of the transplanted kidney in 50% of cases [32,33]. The virus actively multiplies in the renal tubular epithelial cells, and due to cell lysis it enters the distal urinary tract. ‘Decoy cells’ with viral inclusions in the cell nuclei are then detected in the urine. After breaching the tubular epithelial basement membrane, viral particles enter the bloodstream and viraemia occurs.

## 2. Description of BK Polyomavirus

BKPyV is a small, non-enveloped virus with a diameter from 40 to 44 nm and icosahedral symmetry. Its genome consists of about 5000 base pairs and is built of circular, double-stranded DNA [2,14]. The BKPyV genome encodes six major viral proteins and is divided into three functional regions: the early and late coding regions and the non-coding control region (NCCR).

**Figure 1:**
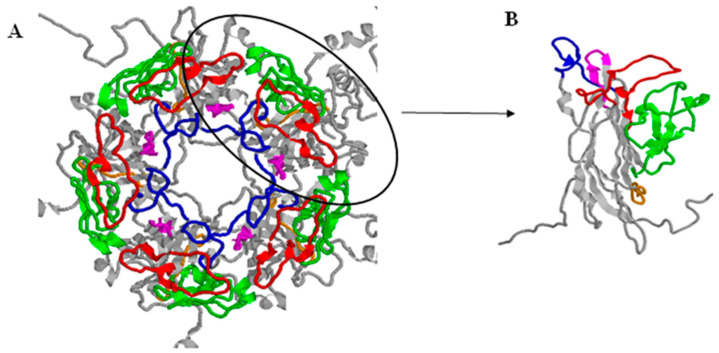
(**A**)—Pentamer of VP1 protein, (**B**)—monomer of VP1. Model based on the sequence obtained and deposited (5fua.1) by [39]. Loop location visualized based on [40]. BC loop–57-89aa–red, DE loop–129-147aa–blue, EF loop–157-218–green, GH loop–247-257aa–orange, HI loop–268-277–magenta. Visualization was prepared in Rasmol Software. Adapted from [39].

The early coding region consists of two open reading frames, which are formed (each on separate mRNA) after alternative splicing of the one early transcript. The early coding region contains genes coding for two non-structural proteins: large (TAg) and small (tAg) tumour antigen [26,34,35]. The late coding region is transcribed after the start of virus genome replication. It codes for three viral capsid proteins, VP1, VP2 and VP3, and a non-structural agnoprotein. The major capsid protein VP1 can be divided into five outer loops, BC, DE, EF, GH and HI, which connect the various β-strands of the polypeptide (Figure 1). In the shallow groove between the BC and HI loops there is a receptor binding site, while the BC loop contains the region with nucleotides at positions 1744–1812, used to identify the four main BKPyV genotypes on the basis of nucleotide polymorphisms [36]. The agnoprotein plays a role in many cellular processes, including cell cycle progression, transcription regulation of the virus, capsid construction, inhibition of host DNA repair function, and virion release from the cell [9,37,38].

Between the early and late regions, there is a hypervariable non-coding control region (NCCR), also called a regulatory region [38]. The NCCR contains cis-acting elements, i.e., regulatory sequences within promotors and enhancers, the origin of DNA replication (ori), and binding sites of T antigen and cellular transcription factors [41]. Transcription factors regulate gene expression in both the early and late region [38]. The NCCR is divided into five blocks: block O, which contains the origin of replication, and four sequence blocks binding transcription factors—P, Q, R and S [42]. Point mutations, deletions, duplications and rearrangements in the NCCR cause different strains of BKPyV to exhibit considerable heterogeneity within it, and this variation most likely plays a key role in the virus replication rate.

The P, Q, R, and S blocks of NCCR constitute the transcriptional control region (TCR), which harbours a number of cellular transcription factor binding sites. This genome segment is particularly prone to mutations. It was proposed that rearrangements in NCCR may change transcription factor binding sites and, in that way modulate the replication rate [43]. Increased viral replication may be also caused by the deletions of putative inhibitory sequences within the Q and R blocks. However, there is also possibility that the enhanced virus replication rate in immunosuppressed individuals leads to greater molecular diversity of BKPyV. The results obtained by Liimatainen et al. 2020 indicate that immunosuppression may trigger increased viral replication, which may lead to the mutations in the transcriptional control region and the emerging of new virus variants displaying enhanced replication efficiency [44].

Rearrangements are believed to allow the virus to adapt to changes taking place in the host cell environment by increasing or decreasing the number or affinity of transcription factor binding sites [41]. Although rearrangements in the case of BKPyV may be sporadic rather than specific, the consequently enhanced replication rate is probably associated with the increased rate of clinical disease.

## 3. Molecular Polymorphism of the BK Polyomavirus

Based on single nucleotide polymorphisms (SNPs) in the viral protein VP1 region and the non-coding control region (NCCR), the virus has been divided into genotypes and subtypes [26]. The first classification of strains into genotypes was described in 1993 by Jin et al. and was based on the sequence and the restriction sites specific for a given genotype in the variable region of the gene encoding the VP1 protein [45,46]. The VP1 coding region shows very high similarity (over 95%) in all genotypes of BKPyV, but the similarity between the amino acid residues from 61E to 83R is only 61–70% [35].

Sixty polymorphic sites have been identified in the complete coding sequence of the VP1 protein of the BK polyomavirus (1089 base pairs), based on which isolates can be differentiated and assigned to a specific subtype [47]. Four main genotypes of the virus have been distinguished among BKPyV isolates: I, II, III and IV. Groups I and IV are heterogeneous, and therefore have been further divided into subgroups. Four subtypes have been distinguished within genotype I (Ia, Ib-1, Ib-2 and Ic) and six within genotype IV (IVa-1, IVa-2, IVb-1, IVb-2, IVc-1 and IVc-2), for a total of 12 different BKPyV variants [47,48].

The most common BKPyV type worldwide is genotype I (about 80% of recorded cases), followed by genotype IV (about 15% of recorded cases), while genotypes II and III are the least frequently encountered. The distribution of molecular variants of the virus is associated with the region of origin. Subtype Ia is the most common subtype in Africa, Ib-1 is widespread in Southeast Asia, Ib-2 in Europe, and the most common subtype in Northeast Asia is Ic [35,47,48,49]. The subtype of the virus identified in a patient can thus provide information on the ethnic origin of the individual the sample was taken from [48,50,51,52,53].

## 4. Pathogenesis of BKPyV Infections

Infection with the BK polyomavirus takes place in early childhood [54]. Primary infection is usually asymptomatic, and when symptoms do occur, they are mainly fever, upper respiratory symptoms, and transient cystitis [1,55]. The route of transmission of the virus is not precisely known, but based on the symptoms it induces, transmission via the urine or respiratory tract is most commonly postulated [9,26,32]. Following primary infection, the virus replicates in the respiratory tract, where it can cause transient symptoms. It has been suggested that the virus enters the bloodstream via infected tonsils and subsequently infects peripheral blood mononuclear cells and with them enters other organs, including the kidneys [56]. BKPyV exhibits tropism for the renal tubules and cells of the transitional epithelium of the urinary tract, in which it becomes persistent [8].

### 4.1. BKPyV Infection in Kidney Transplants Recipients

The literature describes cases of transmission of the BK polyomavirus in the kidney from a seropositive donor to a seronegative recipient during kidney transplantation [57,58]. In addition to the transmission routes mentioned above, the virus can be transmitted during blood transfusion and with semen [9,59]. Research has also shown that BKPyV can penetrate the placental barrier, which confirms vertical transmission of the pathogen [60].

In immunocompetent individuals, the presence of BKPyV is usually asymptomatic. BKPyV has been recognized as the cause of disease in the native kidneys of patients with AIDS-induced immunodeficiency and in patients undergoing cancer treatment. In transplant recipients, in whom the immune response process is impaired by treatment with immunosuppressant drugs, the virus may replicate, leading to serious complications. However, the mechanism of the persistence of the virus and the conditions leading to its replication following immunosuppression remain unclear [2,8]. The main complications resulting from the presence of BKPyV in this group of patients are BK polyomavirus nephropathy (BKPyVAN), ureteral stricture, and haemorrhagic cystitis, which can lead to kidney failure [9,61].

The fact that BKPyV replication in kidney transplant recipients is essentially due to the donor strain was suspected for a long time, since they are much more susceptible to BKPyV replication and disease than other SOT with stronger immunosuppression. Schwarz et al. 2016 compared the virus genotype present in donors and recipients before and after transplantation. Authors showed that the virus genotype detected in the recipient after the transplant differed from the genotype present before the transplant, but was fully concordant with the genotype of the virus in the donor and detect donor-recipient transmission in 24 of the 28 corresponding pairs [62]. The study provided evidence of the importance of infection with the virus during transplantation, but did not show whether the origin of the virus affected the frequency of development of BKPyVAN [62]. Similar conclusions have been drawn by Schmitt et al. 2014, as they noticed that the BKPyV variants found to replicate in kidney transplants recipients correspond in most cases to those ones detected in the respective donors [63]. Additionally, Hirsch and Randhawa 2019 reported that such conditions as BKPyVAN and organ-invasive disease occur most frequently in kidney transplant, but rarely in non-kidney SOT [64].

A symptom of active replication of the virus in transplant recipients is excretion of the virus in the urine, i.e., viruria (80% of patients), while viraemia, where virions are detected in the blood, appears in 10–15%, and severe tubulointerstitial nephritis (BKPyVAN) occurs in about 8% [14,26]. In some patients with BKPyVAN irreversible kidney damage takes place, resulting in loss of the transplant [32,65,66]. BKPyVAN is the most serious and best described condition induced by the BK polyomavirus. In states of immunosuppression, BKPyV can replicate in the epithelial cells of the renal tubules, causing necrosis and lytic destruction with denudation of the basement membrane. This enables accumulation of tubular fluid in the interstitial space, which causes interstitial fibrosis and tubular atrophy, leading to damage to structural elements (nephrons) and consequently to the failure of the transplanted kidney [14]. It remains largely unknown whether there are differences between the causal roles of the various BKPyV subtypes in the development of clinical syndromes and nephropathy, and available reports indicate that infection with genotype IV of the BK polyomavirus may be linked to a higher risk of BKPyVAN [46,62].

### 4.2. BKPyV Infection after Allogeneic Haematopoietic Stem-Cell Transplantation

While severe tubulointerstitial nephritis (BKPyVAN) and ureteral stricture appear most often in kidney transplant recipients, BKPyV infection following allogeneic haematopoietic stem-cell transplantation (HSCT) is usually associated with haemorrhagic cystitis (HC) [35,67,68,69]. HC is the most serious consequence of virus replication and develops in 5–25% of recipients of allogeneic HSC transplantations [46,70,71,72]. HC is characterized by haemorrhaging of the bladder mucosa with painful urination. The severity of the condition can range from minor haematuria (grades 1 and 2) to the formation of clots in the bladder, ultimately resulting in native kidney failure (grades 3 and 4) [9].

There are no conclusive data on the occurrence of haemorrhagic cystitis in relation to the mortality rate of HSCT recipients. Gilis et al. 2014 found no correlation between mortality and the occurrence of this disease entity, while emphasizing that complications in the form of haemorrhagic inflammation were linked to longer hospitalization and higher financial expenditure for treatment [73]. Similarly, Lunde et al. found no difference between the overall survival rate at 1 year between a group with HC (63%) and the control group (66%) [74]. In contrast, Cesaro et al. 2015, in a study in 107 paediatric patients with HSC transplantations, found that mortality was significantly higher in the group with HC [75]. This confirmed the findings of Cesaro’s previous research, in which the survival rate was lower in the group of paediatric HSCT recipients in which HC had developed (overall survival—40% in group with HC vs. 65% in group without HC) [76].

Origin of BKPyV in the case of HSCT is not quite conclusive, there are several proposed scenarios. This may be due to the immunosuppression which triggers replication of the persistent BKPyV. Another possibility is that replication may be induced by the mutation or rearrangements in NCCR region or alternatively viral replication may be result of the transmission of BKPyV from the donor to the recipient [77]. There are also studies suggesting replication of BKPyV as a result of the nosocomial transmission via medical personnel, medical instruments, or common toilets [78,79].

A characteristic trait of BKPyV infection is viruria, which can be present in more than 60% of people with impaired immune function [33]. Replication has also been observed in pregnant women and in a group of elderly patients with diabetes [70,80]. Hirsch reported that asymptomatic viruria may appear in about 5% of healthy individuals [32,33], while a study by Dehchesmeh showed that the current frequency of BKPyV viruria may be even higher, reaching nearly 13% in the population tested [31].

### 4.3. Oncogenic Potential of BKPyV

Some studies suggest that BKPyV infection may be a significant risk factor for the development of bladder cancer in both immunocompromised people and those with normal immunity [81,82]. The precise role of BKPyV in carcinogenesis remains in dispute, but the TAg antigen is known to be capable of binding to suppressor proteins pRB and p53, which deactivates them and initiates the cell cycle in host cells [37]. Deactivation of pRB causes the cell to enter the proliferation process, while binding of TAg to p53 inhibits apoptosis. This leads to conditions conducive to carcinogenesis [83,84].

TAg has also been shown to activate the DNA methyltransferasy1 gene, causing hypermethylation of tumour suppressor genes, which facilitates their inactivation [85]. In experiments in mice, inactivation of pRB and p53 by TAg in the epithelium of the urinary tract caused a tumour reminiscent of cancer in humans [78]. Moreover, TAg has been detected in bladder cancer cells in transplant patients, and a correlation has been demonstrated between a large number of BKPyV viral particles in the urine and cases of bladder cancer [81].

Some research results indicate that chronic BK polyomavirus infections may cause the virus to integrate with the host genome and lead to overexpression of the viral Tag protein in immunocompromised individuals. This is an oncogenic factor due to its effect on the p53 protein and impairment of the expression of the host genes [86]. Integration of BKPyV into host genome seems to be relatively rare event, as it is described in only few papers in a small number of patients (two cases in studies of Jin et al. 2021, three cases in studies of Wang et al. 2020, one case in the study of Muller et al. 2018, one case in the study of Kenan 2017, and one case in the study of Kenan 2015) [86,87,88,89,90].

### 4.4. Therapeutic Management

The important issue concerning BKPyV is therapeutic management. This topic is comprehensively presented in “BK polyomavirus in solid organ transplantation—Guidelines from the American Society of Transplantation Infectious Diseases Community of Practice”. The guidelines provide recommendations concerning the frequency of BKPyV-DNAemia screening in kidney transplant recipients, the algorithm of therapeutic management and strategies of BKPyV-DNAemia and BKPyVAN treatment. Presently, there are no randomized controlled trials confirming that adjunctive use of such drugs as leflunomide, cidofovir, fluoroquinolones, or intravenous immunoglobulin (IVIG), is more advantageous than a reduction in immunosuppression alone. As there is no casual treatment, modulation of immunosuppression plays a crucial role in the reduction in viral replication. Therefore, the guidelines recommend reducing the strength of immunosuppression. Depending on the expected and achieved therapeutic effect, a detailed multi-stage procedure is most often recommended, consisting of reducing the dose of a calcineurin inhibitor by 25% to 50% and/or reducing the antiproliferative drug initially by 50% until its complete discontinuation. There are also additional strategies, which recommend switching from tacrolimus to low-dose cyclosporine-A, or using sirolimus instead of calcineurin inhibitors, or replacing mycophenolic acid with low-dose sirolimus, or mycophenolic acid with leflunomide [64].

## 5. Methods of BK Polyomavirus Diagnosis

Replication of a virus is diagnosed on the basis of the presence of its genetic material beyond the site where it remains persistent. In the case of BKPyV, the site of persistence is the kidneys, and a sample of the patient’s urine or blood is usually tested [14]. Detection of the virus is not synonymous with the onset of disease, but the number of virus particles present in the sample must be regularly monitored, because its presence in the urine and especially in the blood of an immunocompromised patient always carries the risk of further serious complications, which can lead to the destruction of the transplant [32,57,91]. Virally infected epithelial cells with basophilic intranuclear inclusion bodies (‘decoy cells’) can be detected by urine cytology, but because these cells do not conclusively indicate BKPyVAN, but only active replication of the virus in the urinary tract, the diagnostic value of this method is much lower [3,9,32].Decoy cells alone do not confirm BKPyV infection, as they are not specific for the presence of BKPyV in urine and can be found, for example, in the case of JC polyomavirus and adenovirus infections [92]. Therefore, a definitive diagnosis of BKPyVAN requires a biopsy [8].

### 5.1. PCR

There are many methods for identifying and characterizing viruses. One of the methods commonly used to detect BKPyV in medical and biological laboratories is polymerase chain reaction (PCR). PCR is a molecular technique enabling enzymatic amplification of a selected DNA or RNA sequence [93]. In the case of BKPyV, PCR is usually performed to amplify highly conserved sequences in genes coding for the major protein VP1, TAg or NCCR [94,95,96]. The most important precondition for performing a polymerase chain reaction is knowledge of the sequences of the regions flanking the target region. This information is essential for designing the pair of primers that anneal to the strands in complementary sites and enable amplification of the desired fragment (Supplementary Materials Figure S1).

The method is based on exploitation of the ability of DNA polymerase to synthesize a new strand complementary to the available template strand. The reaction has three main stages: denaturation of double-stranded DNA to separate the complementary strands, annealing of primers to the DNA template, and synthesis of a chain (elongation) by thermostable DNA polymerase, which adds free nucleotides present in the solution, serving as building blocks for new strands, to the sequence. These stages are repeated cyclically, and in each successive cycle the DNA fragments synthesized in the previous cycles serve as the template. Then the reaction mixture is subjected to electrophoresis to determine whether a fragment of the expected size has been amplified, and thus whether the infectious agent is present in the sample (Supplementary Materials Figure S2).

Since the technique enables amplification and detection of even a small number of copies of the pathogen, it can be used to detect the virus at a very early stage of disease, when it is still present in a small amount, thus allowing treatment to be initiated immediately. PCR causes an exponential increase in the number of copies of the reaction products. In theory, assuming 100% yield of the reaction, after n cycles 2^n^ copies can be obtained from one copy of the template.

PCR is also a preliminary technique for further analyses, such as sequencing and subsequent bioinformatic analysis, which enable phylogenetic analysis or functional analyses of genes [97,98]. An additional advantage of PCR is that it can detect the virus in original tissue, so that mutations associated with adaptation to a cell culture can be avoided. Detection of the BK polyomavirus in the urine is a rapid and non-invasive means of identifying the virus in patients at risk of BK nephropathy and other complications arising from its presence, as well as monitoring the response to treatment [99]. However, conventional PCR has some limitations and drawbacks. To confirm the presence of a specific product of the reaction in the sample, it is necessary to prepare electrophoretic separation of the amplicons. This additional step makes analysis more prone to contamination and more time-consuming. To reduce the risk of occurrence of false positive and false negative results it is necessary to stick to the GLP principles and to include positive and negative control in each step of the analysis.

### 5.2. qPCR

As BKPyVAN develops asymptomatically, the most effective strategy for monitoring viruria and viraemia (BKPyV-DNAemia) and for early diagnosis and treatment of BK nephropathy is regular monitoring of the viral load through quantification of viral DNA by quantitative PCR (qPCR) [26]. Additionally, quantification of BKPyV mRNA (usually BKPyV VP1 mRNA) enables detection of ongoing viral transcription [100].

The difference between the classic and quantitative methods is that in qPCR the increase in product is monitored in real time (Supplementary Materials Figure S3). Fluorescent dyes or specially labelled probes that emit a fluorescent signal proportional to the amount of product are used in the reaction, and measurement of this signal is used to monitor the increase in product and to quantity the viral load in the sample [96]. The number of copies is determined by referring to a standard curve plotted from standards with a known number of copies. The qPCR method, apart from diagnosis of BKPyV, is used to monitor the effectiveness of treatment, making it possible to determine the number of viral particles present in the biological samples such as plasma or urine [8]. Another important advantage of qPCR is that it eliminates the electrophoresis stage needed in classic PCR, thus reducing the duration of the entire process and the possibility of contamination is lower. BKPyV should be monitored by qPCR until the level of the virus can no longer be detected or falls to below the threshold value that can lead to nephropathy [101].

Guidelines from the American Society of Transplantation Infectious Diseases Community of Practice emphasizes the meaning of evaluation of BKPyV-DNA in plasma and urine, as valuable prognostic biomarker. However, there are the difficulties in obtaining comparable quantitative viral data internationally. To address these issues and to ensure comparability, proper standardization and validation of BKPyV-QNAT assays (quantitative nucleic acid amplification testing) are necessary. It can be achieved by such improvements as development of BKPyV calibrator approved by WHO (World Health Organization). Moreover, diagnostic laboratories should participate in proficiency testing provided by national and international external quality assurance programs to reduce interlaboratory variation of the results (stemmed from the technical variation related to DNA extraction, cycling conditions and the variability of the viral genotypes) [64].

### 5.3. Genotyping

**Figure 2:**
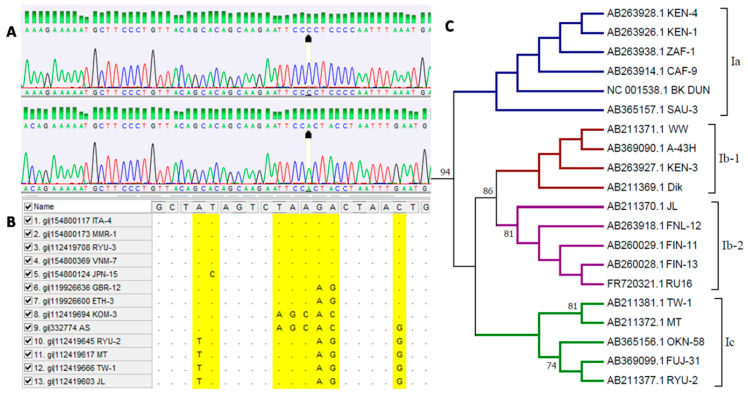
Bioinformatic analysis of BK polyomavirus sequences including (**A**) analysis of chromatograms representing sequences belonging to BKPyV genotypes I and IV (analysis performed in DNA Baser software), (**B**) preparation of sequence alignment from databases representing different virus genotypes, (**C**) analysis of phylogenetic relationships between virus subtypes within genotype I (analysis performed in MEGA 6 software). Own results.

For a molecular characterization of BKPyV, analysis of the distribution of the variants of the virus in a given population, and determination of phylogenetic relationships between isolates, it is essential to identify the genotypes of the virus (Figure 2). A common method of genotyping BKPyV is sequencing [35,47,48,49,53,102]. The first genotyping scheme for BKPyV was described by Jin et al. in 1993 and was based on nucleotide polymorphisms in a very short fragment of the gene of the capsid protein VP1 (nucleotides 1744 to 1812), which were detected by restriction fragment length polymorphism (RFLP). The authors established that BKPyV shows genotype variation, based on which four main genotypes of the virus (I–IV) can be distinguished [103]. With the advancement of research and knowledge of the genetic variation in BKPyV, the sequencing reaction was used for further division into subtypes of the virus [47].

The complete genome of the BK polyomavirus was sequenced and published in 1979 [104], and the NCBI database currently contains more than 4000 nucleotide sequences belonging to the virus. This is due in part to the fact that recent years have seen the rapid development of next generation sequencing (NGS) methods, leading to an improvement in the molecular methods used [3,26]. NGS is a powerful tool, which gives more precise information concerning the virus population of the patient. Liimatainen et al. 2020, used NGS to detect and analyse rearrangements in the transcriptional control region (TCR) of BK virus [44]. NGS may also be used in metagenomic analysis, such an approach enables not only the analysis of BKPyV but also to determining the presence and abundance of transplant-related viral infections [105].

However, Sanger sequencing is still used successfully for BKPyV genotyping [47,53] due to the relatively short length of the sequence used to classify the virus into subtypes. BKPyV genotyping still relies mainly on identification of polymorphisms within the complete sequence encoding the VP1 protein of the BK polyomavirus, because many studies based on this region have made it possible to learn the variation in it and to develop schemes based on which a given isolate can be assigned to a specific subtype (Table 1).

However, research is increasingly conducted to identify polymorphisms and enable classification of BKPyV based on variation in regions of the large T antigen and NCCR [47,95]. Sequencing is an extremely important method making it possible to identify the BKPyV genotypes circulating in a population and provides information on genotypic variation, the distribution of variants around the world, and initial geographic location, enabling the formulation of hypotheses on the evolution and migration of the virus [51,52,53]. Knowledge of the virus genotypes is also essential for implementing and updating diagnostic tests, studying the immune response to infections with different BKPyV variants, and potentially designing vaccines to stimulate antibody production and improve the prognosis of immunocompromised patients infected with the BK polyomavirus [103,106]. Molecular polymorphism, especially in the BC loop of the VP1 protein, triggers synthesis genotype-specific antibodies and make it possible to escape from neutralization by antibodies raised against the other types [95]. Therefore, genotype-specific antibodies are demanded to introduce proper immunotherapeutic strategy.

Bioinformatic analysis also makes it possible to analyse the effect of polymorphisms in the nucleotide sequence on the amino acid sequence and functionality of a protein. Single nucleotide polymorphisms (SNPs) are believed to be important in the pathogenesis of the virus. Varella et al. 2018 found differences in viral load between the Ia and Ib2 subtypes, which differ in the sequences coding for VP1 and in the NCCR [107]. Single changes in the nucleotide sequence can affect not only its pathogenicity but also the tropism of the virus, or the range of hosts. In the case of the JC polyomavirus, nonsynonymous mutations in the BC and HI loops of the VP1 protein may be linked to the occurrence of progressive multifocal leukoencephalopathy (PML). Among 20 patients with PML, JCPyV variants containing mutations leading to changes in the amino acid sequence of the VP1 protein were isolated from 17 [108,109]. Rearrangement of NCCR of JCPyV, may also play an important role in pathogenesis of PML, as JCPyV variants carrying rearranged NCCR were usually isolated from PML patients [108]. JCPyV variant with NCCR rearranged exhibited high viral replication and a wide host cell susceptibility in comparison to the JCPyV with NCCR archetype form [110].

In the case of the BKPyV as well, higher molecular variation has been observed in variants isolated from patients with BKPyVAN than from healthy donors or recipients in which the transplant was not destroyed [111]. The authors suggested that the genetic instability of the virus may contribute to the pathogen’s ability to avoid the host immune response, resulting in an increase in resistance to antiviral drugs. Luo et al. (2011) also confirmed high variation in the BC loop of the BK polyomavirus, leading to the occurrence of a quasispecies that may be associated with higher pathogenicity. McIlroy et al. noted that in kidney recipients with BKPyV replication, the wild variant was initially dominant in the virus pool, but rapid replication resulted in variants with mutations in the BC loop of the VP1 protein, which may affect parameters such as infectivity and resistance to neutralization by antibodies [112]. There are reports confirming that the virus may exploit the host to increase the mutation rate in the VP1 protein. Verhalen et al. showed that the BK polyomavirus can induce expression of the host’s *APOBEC3B* (*A3B*) gene (apolipoprotein B MRNA editing enzyme catalytic subunit 3B–DNA editing cytidine deaminase enzyme) [113], and, according to Peretti et al., the pathogen can even use the host *A3B* to acquire mutations that help it to escape neutralizing antibodies [114]. Higher molecular variation in the case of patients with BKPyV, may also be caused by the enhanced replication leading to higher possibility of severe disease and tissue damage. Boosted viral replication after prolonged immunosuppression may lead to accumulation of mutations. Therefore, enhanced replication may be not only the result but also the cause of the higher molecular variation of BKPyV [44].

**Figure 3:**
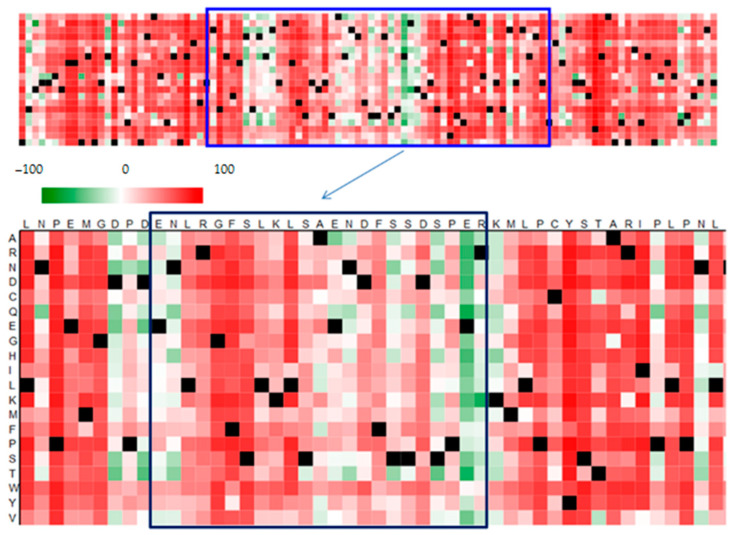
Prediction of functional effects of mutations in the amino acid sequence of the VP1 protein with a BC loop region (in frame) prepared in the SNAP2 application, showing polymorphic amino acids in relation to the DUN strain. Dark red indicates a high score (score > 50, strong signal for effect), white indicates weak signals (−50 < score < 50) and green a low score (score < −50, strong signal for neutral/no effect). Black designates the corresponding wild-type residues. Own results.

Given the significant functional effect of polymorphisms, bioinformatic methods can be an excellent tool for predicting and modelling the potential effects of nonsynonymous mutations in the genome of the virus (Figure 3). Bioinformatic tools are associated with databases such as NCBI or ENSEMBL, which provide a platform for exchanging and gathering data on nucleotide and amino acid sequences. Access to sequences in databases minimizes the need to test reference samples, because comparison of the sequence obtained with database resources enables conclusive identification of the pathogen, as well as comparison of the variant with isolates from various parts of the world.

## 6. Conclusions

The BK polyomavirus is widespread in the contemporary population, which is confirmed by the presence of anti-BK antibodies in most populations tested. The virus remains persistent in immunocompetent individuals, and its replication occurs in the case of immunosuppression. For this reason, the BK polyomavirus is a significant problem in organ transplantation and necessitates a compromise between inhibiting replication of the pathogen and maintaining the transplant.

Although the problem of BKPyV infections has been studied since the 1970s, there are still many unknowns regarding the mechanisms by which the virus penetrates the cell, mechanisms of persistence, replication, and geographic specificity of BKPyV. Recent achievements, including advancements in genomic techniques, have contributed to a much better understanding of the course of infection and the molecular epidemiology of BKPyV. Nevertheless, the epidemiology of BKPyV is a complex question requiring an interdisciplinary approach, involving not only doctors of various specializations (urologists, transplant surgeons or nephrologists), but also integration of knowledge in the areas of genetics, biotechnology, and bioinformatics.

## Figures and Tables

**Table 1:** Variable region in VP1 protein of BKPyV ranging from 61aa to 82aa (amino acids).

		Variable Region										
Genotype/Subtype	Accession Number	61	62	66	68	69	71	73	74	75	77	82
Ia	NC_001538.1_BK_DUN	E	N	F	L	K	S	E	N	D	S	E
	AB263926.1_KEN-1	.	.	.	.	.	.	.	.	.	.	.
Ib-1	AB211371.1_WW	.	.	.	.	.	.	K	.	.	.	.
	AB211369.1_Dik	.	.	.	.	.	.	.	.	.	.	.
Ib-2	AB260029.1_FIN-11	.	.	.	.	.	.	.	.	.	.	D
	AB260028.1_FIN-13	.	.	.	.	.	.	.	.	.	.	D
	AB211370.1_JL	.	.	.	.	.	.	.	.	.	.	.
Ic	AB211381.1_TW-1	.	.	.	.	.	.	.	.	.	.	.
	AB211377.1_RYU-2	.	.	.	.	.	.	.	.	.	.	Q
	AB211372.1_MT	.	.	.	.	.	.	.	.	.	.	.
II	AB263916.1_ETH-3	.	.	Y	.	.	T	.	.	A	D	D
	JN793996.1 KT40	D	.	Y	.	.	T	.	.	A	D	D
	AB301101.1 J2B-11	D	.	Y	.	.	T	.	.	A	D	D
III	M23122.1_AS	D	.	Y	Q	H	.	.	.	A	E	D
	JN192440.1 SJH-LG-310	.	.	Y	Q	H	.	.	.	A	E	D
	AB365139.1 NEA-27	D	H	Y	Q	H	T	.	.	A	D	D
	AB365130.1 FUK-22	D	H	Y	Q	H	T	.	.	A	D	D
IV	AB211388.1_KOM-7	N	D	Y	.	R	T	.	T	A	D	D
	AB211387.1_KOM-2	N	D	Y	.	R	T	.	T	A	D	D
	AB211391.1_TW-3	N	D	Y	.	R	T	.	T	A	D	D
	AB211390.1_THK-8	N	D	Y	.	R	T	.	T	A	N	D
	AB211389.1_RYU-3	N	D	Y	.	R	T	.	T	A	D	D

## Supplementary Materials

The following are available online at https://www.mdpi.com/article/10.3390/v13081502/s1. Figure S1: Diagram illustrating the PCR design, indicating the conserved sites where the primers bind to the sequence encoding the VP1 protein and the flanking sequence containing polymorphic nucleotides used to classify isolates of the virus into genotypes and subtypes. Analysis and visualization prepared in MEGA6 Software with data retrieved from GenBank. Own results. Figure S2: Electrophoretic separation of amplification products obtained in a reaction with primers flanking the fragment encoding the VP1 protein of the BK polyomavirus. The presence of a light 327 bp band indicates the presence of the genetic material of the virus in the sample. Separation was carried out in 2% agarose gel. Own results. Figure S3: qPCR, allowing for quantification of the viral load in the sample, together with the standard curve based on amplification of four standard samples. qPCR amplification prepared with the GeneProof BK/JC Virus (BK/JC) PCR Kit. Own results.
