# Supplementary figures and images for: BK Polyomavirus—Biology, Genomic Variation and Diagnosis
Source: Viruses. 2021 Jul 30;13(8):1502. doi: 10.3390/v13081502 (PMC8402805; doi:10.3390/v13081502)

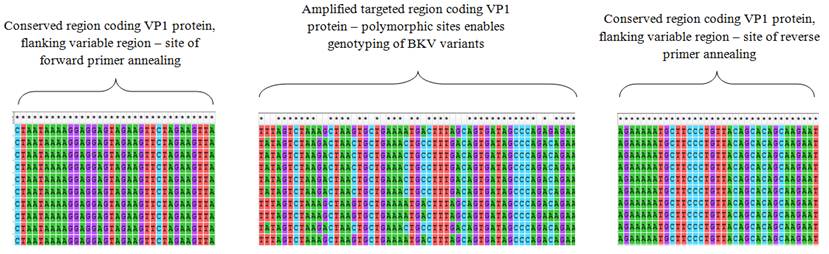

Supplement: Supplementary file 1 [file viruses-13-01502-s001.zip › Figure S1.jpg]

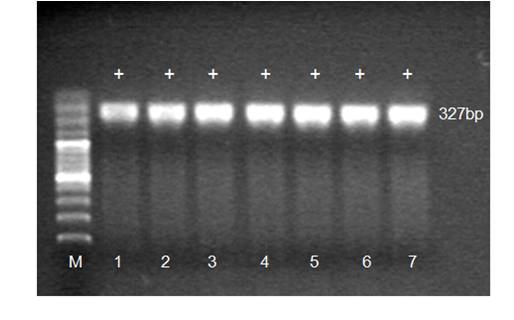

Supplement: Supplementary file 1 [file viruses-13-01502-s001.zip › Figure S2.jpg]

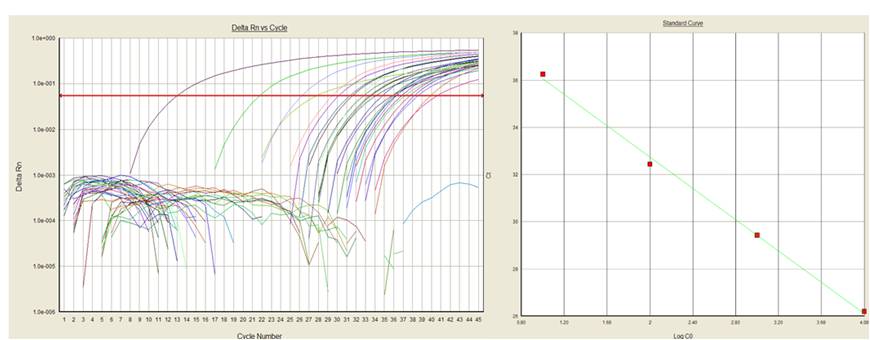

Supplement: Supplementary file 1 [file viruses-13-01502-s001.zip › Figure S3.jpg]
